# Difference DV_Distance Localization Algorithm Using Correction Coefficients of Unknown Nodes

**DOI:** 10.3390/s18092860

**Published:** 2018-08-30

**Authors:** Lijun Sun, Tianfei Chen

**Affiliations:** School of Electrical Engineering, Henan University of Technology, Zhengzhou 450001, China; sunlijunzz@163.com

**Keywords:** wireless sensor network, node localization, DV_Distance, unknown node, difference, distance correction

## Abstract

Node localization is an essential requirement in the increasing prevalence of wireless sensor networks applications. As the most commonly used localization algorithm, the DV_Distance algorithm is more sensitive to ranging error, and also has lower localization accuracy. Therefore, this paper proposes a novel difference DV_Distance localization algorithm using correction coefficients of unknown nodes. Taking account of the fact that correction coefficients of unknown nodes should be different, the proposed method has employed the correction model based on unknown nodes. Some correction coefficients for different direction anchor nodes can be indirectly calculated using the known difference of actual Euclidean distance and corresponding accumulated hop distance between anchor nodes, and then the weighting factors for the correction coefficients of different direction anchor nodes are also computed according to their actual contribution degree, so as to make sure that the corrected distances from unknown nodes to anchor nodes, modified by the final correction coefficient, are closer to the actual distances. At last, the positions of unknown nodes can be calculated using multilateral distance measurement. The simulation results demonstrate that the proposed approach is a localization algorithm with easier implementation, and it not only has better performance on localization accuracy than existing DV_Distance localization algorithm, but also improves the localization stability under the same experimental conditions.

## 1. Introduction

In recent years, the advantages of wireless communication technology, sensors, and embedded technology have significantly propelled the development of wireless sensor networks (WSNs) which have already been widely applied to some key fields [1,2], such as environmental monitoring, traffic management, national defense, and so on, in order to achieve real-time acquisition and perception of various object information in monitoring areas. Generally, the smart sensor nodes are often randomly and unevenly distributed, and the topology of WSNs may sometimes change dynamically, and thus it is difficult to determine the positions for all the sensor nodes. But, the position information of sensor nodes is indispensable and the monitoring without position information is also meaningless. Besides that, the position information of sensor nodes can help design the top-level route protocols [3,4,5]. Therefore, localization algorithm for sensor nodes is one of the key technologies is WSNs [6]. 

**Figure 1:**
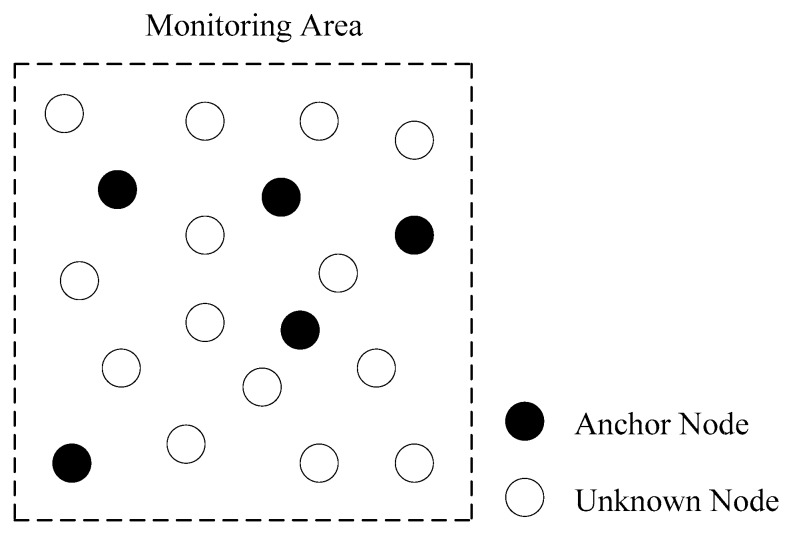
An example of wireless sensor networks (WSNs).

WSNs consist of a large number of sensor nodes, and the Figure 1 shows an example of network structure. Each node can be classified as either an unknown nodes or as an anchor node. Traditionally, anchor nodes can be localized by using Global Positioning System (GPS) or BeiDou Navigation Satellite System (BDS). However, it is impossible for the use of GPS/BDS on each node, because it requires higher hardware or deployment costs, which are not acceptable in large-scale WSNs. Therefore, there are only a few of anchor nodes compared to the number of unknown nodes in WSNs, and most unknown nodes have to estimate distances from anchor nodes over multi-hop environments. When the positions of anchor nodes in WSNs are already given, Node localization refers to the process of computing the positions of unknown nodes after mathematical or physical model have been established through information exchange and calculation. 

At present, there are many kinds of self-localization algorithms, which can be divided into two categories, that is, range-based localization algorithm [7] and range-free localization algorithm [8]. Range-based algorithms use absolute point-to-point distance or angle information to calculate distance between neighboring sensor nodes, but the range-free algorithms do not need absolute range information. 

In range-free localization algorithms, for example, centroid localization [9], APIT [10], DV_Hop [11], and amorphous [12], nodes have no ability to measure the distances or angles to their neighbors and instead use connectivity or hop information to compute their positions. They are simpler and do not require extra hardware for measurement. The DV_Hop is a typical range-free based localization algorithm due to its simplicity, cost-effectiveness, and robustness, and its basic idea is to calculate the estimated distances by the product of the average distance per hop and the hop numbers from unknown nodes to anchor nodes. On the basis of original DV_Hop algorithm, Lin [13] uses the least square method to modify the average distance per hop between anchor nodes. In reference [14], weighting coefficients are introduced into the correlation matrix of estimated distance, and a new localization algorithm with the weighted hyperbolic localization is proposed. Song [15] proposes an improved weighted centroid DV_Hop localization algorithm, which improves the accuracy after selecting appropriate anchor nodes and replaces maximum likelihood localization by centroid localization, besides that a weighted scheme is also used so that the influence of different anchor nodes can be taken into consideration. In order to further improve localization accuracy, the differential evolution (DE) algorithm, which performance is verified superior to the genetic algorithm (GA) and particle swarm optimization (PSO) algorithm, is used to optimize the weighted square error of the estimated distance [16], so that the global optimal solution can be obtained. Usually, because of unavoidable error in the phase of distance estimation, the localization accuracy of range-free based algorithms is still relatively poor. 

Compared with range-free localization algorithms, range-based localization can provide higher localization accuracy, and the ranging methods obtain information, including distance or angle between sensor nodes, by ranging techniques such as Received Signal Strength Indicator (RSSI) [17], Time-Difference-of-Arrival (TDOA) [18], Time-of-Arrival (TOA) [19], and Angle-of-Arrival (AOA) [20]. TDOA, TOA, and AOA have higher requirements for node hardware, while RSSI is a low power and inexpensive ranging method only relying on the acceptance strength of wireless signals. At present, DV_Distance, proposed by D. Niculesucu and B. Nath [21], is a typical range based node localization algorithm, and RSSI is used to measure the distance between adjacent nodes, and then the actual Euclidean distance can be replaced by the accumulated hop distance. Because the DV_Distance algorithm is sensitive to the ranging error, and meanwhile the accumulated hop distance is generally larger than the actual Euclidean distance, thus localization error comes up inevitably. In order to further reduce localization error, some correction model or coefficients are designed to modify the accumulated hop distance between nodes, so that the corrected distance is more similar to the actual Euclidean distance. 

In this paper, a difference DV_Distance localization algorithm using correction coefficients of unknown nodes is proposed. To the best of knowledge, our contributions are as follows:

(i) The correction model based on unknown nodes is used, so that the unknown nodes can have their own correction coefficients, instead of using the correction coefficients from their nearest anchor nodes, and thus the difference of correction coefficients for unknown nodes can be reflected.
(ii) The difference of accumulated hop distance and actual Euclidean distance between anchor nodes is used to design correction coefficients. Because of no position information of unknown nodes, the distance difference from unknown nodes to anchor nodes cannot be directly computed. Therefore, we use the information of anchor nodes distributed in different direction to indirectly compute the distance difference.
(iii) According to the actual contribution of anchor nodes in different direction, the weighting process is put forward to get the final correction coefficient of unknown nodes, which can further improve the localization accuracy of the proposed algorithm.

The remainder of this paper is organized as follows. In Section 2, we briefly review other researchers’ study on DV_Distance improvement which we have realized. In Section 3, the basic DV_Distance localization algorithm is briefly introduced. We introduce how we refine the DV_Distance algorithm in Section 4. Section 5 illustrates the simulation results, and also we compare and analyze the difference between our proposed algorithm and other improved DV_Distance localization algorithms. Finally, we present our conclusions in Section 6. 

## 2. Related Works

Table 1 presents the description of specific symbols for some improved DV_Distance localization algorithms. In reference [22], Dai introduces both the ratio of actual Euclidean distance to accumulated hop distance and the hops between anchor nodes to design a correction coefficient. However, it still uses the correction model based on anchor nodes, that is, each unknown node receives the corresponding correction coefficient from its nearest anchor node to modify the accumulated hop distance from itself to other anchors. Suppose that, the anchor *i* is the nearest anchor node to the unknown node *k*, and thus the correction equations of Dai’s approach are shown in detail as follows:

(1) $$r_{i,j}=\frac{D_{i,j}}{d_{i,j}}$$

(2) $$cv_{i,j}=\frac{r_{i,j}}{h_{i,j}}$$

(3) $$d_{k,j}^{est}=d_{i,j}-cv_{i,j}\times h_{k,j}$$

In term of the difference of accumulated hop distance and corresponding actual Euclidean distance between anchor nodes, Perkins [23] proposes a difference DV_Distance localization algorithm, and the correction coefficient based on the distance difference between anchor nodes is designed to correct accumulated hop distance from unknown nodes to anchor nodes. However, it has an obvious disadvantage, that is, the accumulated hop distances for unknown nodes on the same path are actually different. The equations for distance correction are as follows:

(4) $$cv_{i,j}=d_{i,j}-D_{i,j}$$

(5) $$d_{k,j.}^{est}=d_{k,j}-cv_{i,j}$$

Taking account of the difference of hop number between anchor nodes, Liu [24] evenly assigns the difference of accumulated hop distance and corresponding actual Euclidean distance to each hop, but the problem ignored in this algorithm is that the assigned distance difference for each hop should also be different when the nodes in WSNs are not evenly distributed. The improved distance correction equations of Liu’s method are expressed as:

(6) $$cv_{i,j}=\frac{d_{i,j}-D_{i,j}}{h_{i,j}}$$

(7) $$d_{k,j}^{est}=d_{k,j}-cv_{i,j}\times h_{k,j}$$

On the basis of Liu’s approach, Wang [25] calculated the average distance difference per hop under unit distance after considering the randomness of node distribution, and the specific correction equations for Wang’s method are as follows:

(8) $$cv_{i,j}=\frac{d_{i,j}-D_{i,j}}{h_{i,j}\times D_{i,j}}$$

(9) $$d_{k,j}^{est}=\frac{d_{k,j}}{1+cv_{i,j}\times h_{k,j}}$$

In the above reference [22,23,24,25], the correction model based on anchor nodes is employed. When the accumulated hop distances from unknown nodes to anchors are to be corrected, the unknown nodes will adopt the corresponding correction coefficients from their nearest anchor nodes, which lead to the phenomena that the different unknown nodes may have the same correction coefficient. Therefore, Shi [26] designed a correction model based on unknown nodes in which the unknown nodes use their own correction coefficients, and a dynamic weighted DV_Distance localization algorithm is proposed using the ratio of actual Euclidean distance to accumulated hop distance between anchor nodes. Different anchor nodes are selected to compute their corresponding correction coefficients, and then a dynamic weighting correction approach is designed to obtain the final correction coefficient. The detailed equations of Shi’s method are expressed as follows:

(10) $$cv_{k,i,j}={\left(\frac{D_{i,j}}{d_{i,j}}\right)}^{\frac{h_{k,i}}{h_{k,i}+h_{k,j}}}$$

(11) $$\omega_{k,i,j}=\frac{{\left(\frac{h_{k,i}}{h_{k,i}+h_{k,j}}\right)}^{-1}}{\sum_{t=1}^{N}{\left(\frac{h_{k,i}}{h_{k,i}+h_{k,t}}\right)}^{-1}}$$

(12) $$d_{k,i}^{est}=\sum_{j=1}^{N}\left(\omega_{k,i,j}\cdot cv_{k,i,j}\right)\times d_{k,i}$$

We have realized the above algorithms by programming with MATLAB so that we can compare our proposed algorithm with them from lots of aspects. The comparison and analysis will be made after we introduce our algorithm in following sections.

## 3. Theoretical Background

**Figure 2:**
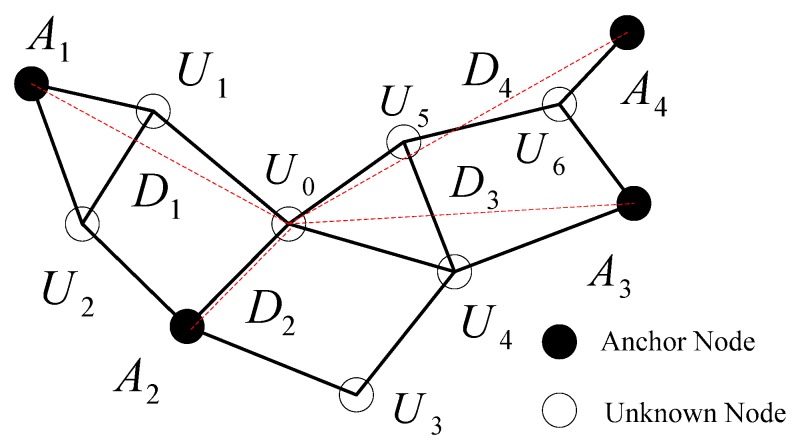
The schematic diagram of basic DV_Distance localization algorithm.

The DV_Distance algorithm is a cooperative localization method based on the distance vector routing principle, and it not only has better distribution and expansibility, but also is easy and simple to be realized. A general WSN is shown in Figure 2, where unknown nodes are represented by white dots, and black dots indicate anchor nodes, besides that, the solid lines between nodes mean that they can communicate with each other, while the dashed lines represent the actual Euclidean distance. In brief, the localization process for the basic DV_Distance algorithm consists of the following two steps: 

Step 1: calculating minimum hop number and corresponding accumulated hop distance from unknown nodes to anchor nodes. 

In terms of the typical distance vector protocol, anchor nodes only broadcast their own information packets, including hop number and accumulated hop distance (both initialized to zero), to neighbor nodes. When an unknown node receives many information packets from a same anchor, it will first compare the hop number and then preserve the information packet having the minimum hop number, after that the hop number will be added 1, and meanwhile the accumulated hop distance from every anchor node will be computed and forwarded to its neighbor node. By means of the above mechanism, both the minimum hop number and the accumulated hop distance from unknown nodes to anchor nodes can be obtained. As shown in Figure 2, the minimum hop number from the unknown node *U*_0_ to anchor nodes *A*_1_, *A*_2_, *A*_3_, and *A*_4_, respectively, are set as 2, 1, 2, and 3, thus the corresponding accumulated hop distances are expressed as *d*_1_ = |*U*_0_*U*_1_| + |*U*_1_*A*_1_|, *d*_2_ = |*U*_0_*A*_2_|, *d*_3_ = |*U*_0_*U*_4_| + |*U*_4_*A*_3_|, and *d*_4_ = |*U*_0_*U*_5_| + |*U*_5_*U*_6_| + |*U*_6_*A*_4_|. In addition, the actual Euclidean distances from *U*_0_ to anchor nodes are represented by *D*_1_, *D*_2_, *D*_3_, and *D*_4_. Obviously, *D*_1_ ≠ *d*_1_, *D*_3_ ≠ *d*_3_, and *D*_4_ ≠ *d*_4_, that is, the accumulated hop distance is not equal to the actual Euclidean distance. 

Step 2: unknown nodes localization using three-edge or multilateral distance measurement.

The accumulated hop distances from each anchor to unknown nodes, which have been calculated in step 1, are regarded as the effective distances, and then the coordinates of unknown nodes can be computed by three-edge or multilateral distance method. Assume that, the coordinates of anchor nodes are (*x_i_*, *y_i_*), *i* = 1, 2, …, *n*, and their accumulated hop distances are respectively *d*_1_, *d*_2_, …, *d_n_*. If the coordinate of unknown node is expressed as (*x*, *y*), we have the equations as follows:

(13) $$\{\begin{array}{c} {\left(x-x_{1}\right)}^{2}+{\left(y-y_{1}\right)}^{2}=d_{1}^{2}\\ {\left(x-x_{2}\right)}^{2}+{\left(y-y_{2}\right)}^{2}=d_{2}^{2}\\ \vdots \\ {\left(x-x_{n}\right)}^{2}+{\left(y-y_{n}\right)}^{2}=d_{n}^{2} \end{array}$$

Then, the Equation (13) can be transformed into the linear equation form of **AX** = **B**, and we have:

$$X=\left[\begin{array}{c} x\\ y \end{array}\right];A=\left[\begin{array}{cc} 2(x_{1}-x_{n}) & 2(y_{1}-y_{n})\\ 2(x_{2}-x_{n}) & 2(y_{2}-y_{n})\\ \vdots & \vdots \\ 2(x_{n-1}-x_{n}) & 2(y_{n-1}-y_{n}) \end{array}\right];B=\left[\begin{array}{c} x_{1}^{2}-x_{n}^{2}+y_{1}^{2}-y_{n}^{2}-d_{1}^{2}+d_{n}^{2}\\ x_{2}^{2}-x_{n}^{2}+y_{2}^{2}-y_{n}^{2}-d_{2}^{2}+d_{n}^{2}\\ \vdots \\ x_{n-1}^{2}-x_{n}^{2}+y_{n-1}^{2}-y_{n}^{2}-d_{n-1}^{2}+d_{n}^{2} \end{array}\right]$$

Finally, the least square method can be used to solve the coordinates **X** of unknown nodes, as shown in Equation (14):

(14) $$X={\left(A^{T}A\right)}^{-1}A^{T}B$$

## 4. Our Proposal: Difference DV_Distance Using Correction Coefficients of Unknown Nodes

At present, the correction model based on anchor nodes is often employed by most existing improved DV_Distance localization algorithms, that is, the accumulated hop distances from unknown node to anchor node is corrected using the correction coefficient of the anchor node which is nearest to the unknown node. Actually, the minimum hop numbers from different unknown nodes to anchor nodes are different, and meanwhile the corresponding accumulated hop distances are also different, and thus different unknown nodes should have different correction coefficients. However, the correction model based on anchor nodes cannot reflect the difference of correction coefficients for different unknown node. 

Taking account of the above technical limitation, a difference DV_Distance localization algorithm using correction coefficients of unknown nodes is proposed, and each unknown node is made to compute its own correction coefficient, reflecting the difference of the correction coefficients for all unknown nodes. Because we have no position information about unknown nodes, it is unable to directly compute the difference of accumulated hop distance and actual Euclidean distance from unknown nodes to anchor nodes. In order to indirectly obtain the distance difference from unknown nodes to anchor nodes, we have defined target anchor node and direction anchor node. For the purpose of explanation, as also shown in Figure 2, anchor *A*_1_ is considered as target anchor node when the accumulated hop distance is modified from unknown node *U*_0_ to anchor *A*_1_, simultaneously, the other anchor nodes *A*_2_, *A*_3_, and *A*_4_ are regarded as direction anchor node. 

The improvement of the proposed algorithm mainly includes two aspects: (1) the contributions of direction anchor nodes are made full use of to compute the distance difference from unknown nodes to target anchor node; (2) according to their contribution, some correction coefficients for all direction anchor nodes are weighted to get the final correction coefficient of unknown nodes, which will further improve the correction accuracy of accumulated hop distance. 

### 4.1. Calculation of Correction Coefficients for Direction Anchor Node

**Figure 3:**
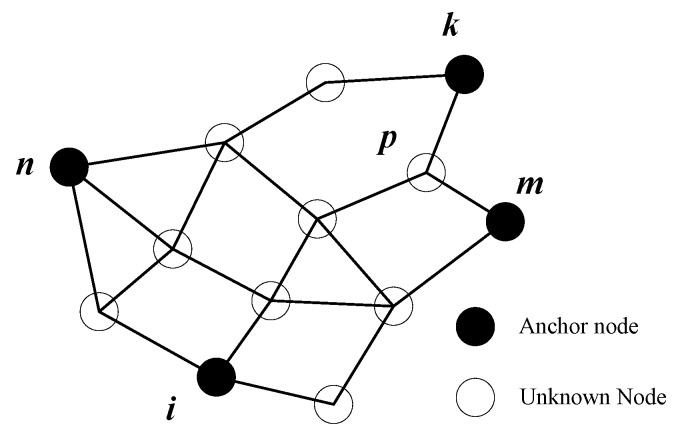
Calculation schematic diagram of correction coefficient.

Unknown nodes do not contain their position information, so the actual Euclidean distances from unknown nodes to anchor nodes are also unknown. Therefore, we put forward an indirect calculation approach for the distance difference from unknown nodes to anchor nodes. Figure 3 shows the calculation schematic diagram of correction coefficients of unknown nodes, node *p* is set as an unknown node, and node *i* is indicated as target anchor node. Both the accumulated hop distance and the hop number from unknown node *p* to target anchor *i* are already known. However, the correction coefficient of unknown node *p* cannot be computed only using the above two known information, and thus, we can make full use of the information from direction anchor nodes *k*, *m*, and *n*. Taking direction anchor node *k* as an example, because the actual Euclidean distance, accumulated hop distance and hop number from direction anchor node *k* to target anchor node *i* are already known, we can calculate the corresponding correction coefficient for direction anchor node *k* using the information from both unknown node *p* and direction anchor node *k* to target anchor node *i*. Similarly, the correction coefficients of other direction anchor nodes can also be obtained sequentially. The calculation equation is as follows:

(15) $$C_{p,i}^{k}=Pc_{p,i}^{k}\cdot \frac{\left(d_{k,i}-D_{k,i}\right)/h_{k,i}}{D_{k,i}}$$

where $C_{p,i}^{k}$ is the correction coefficient for direction anchor node *k* from unknown node *p* to target anchor node *i*; *D_k_*_,*i*_ represents the actual Euclidean distance from direction anchor node *k* to target anchor node *i*; *d_k_*_,*i*_ and *h_k,i_*, respectively indicate the corresponding accumulated hop distance and the hop number. In addition, $Pc_{p,i}^{k}$ is the projection parameter of direction anchor node *k*. 

From Equation (15), it can be seen that the correction coefficient $C_{p,i}^{k}$ for direction anchor node *k* is composed of two parts. In the equation, $\frac{\left(d_{k,j}-D_{k,j}\right)/h_{k,j}}{D_{k,j}}$ indicates the distance difference per hop under unit distance from direction anchor node *k* to target anchor node *i*, and this distance difference can be projected by the remaining part $Pc_{p,i}^{k}$ onto the direction from unknown node *p* to target anchor node *i*. Therefore, the correction coefficients for the other direction anchor nodes can be computed using the same way. 

The projection parameter $Pc_{p,i}^{k}$ can be calculated as follows:

(16) $$Pc_{p,i}^{k}={\left|\cos(angle)\right|}^{\frac{h_{p,k}}{h_{p,i}+h_{p,k}}}$$

where *h_p,k_* and *h_p,i_*, respectively represent the hop number from the unknown node *p* to direction anchor node *k* and target anchor node *i*, and *angle* can be computed in Equation (17) based cosine theorem.

(17) $$angle=\cos^{-1}\left(\frac{d_{p,i}^{2}+d_{k,i}^{2}-d_{p,k}^{2}}{2\cdot d_{p,i}\cdot d_{k,i}}\right)$$

where *d_p,i_* and *d_p,k_*, respectively indicate the accumulated hop distance from unknown node *p* to target anchor *i* and direction anchor *k*. 

For the Equation (17), a triangle can be formed by unknown node *p*, target anchor node *i* and direction anchor node *k*. because we do not have the coordinates of unknown node *p*, and thus the actual Euclidean distances between them can be approximately replaced by the corresponding accumulated hop distances. When the *angle* is 0 degree, it indicates that the shortest path from direction anchor node *k* to target anchor node *i* passes through the unknown node *p*. However, when the angel is 180 degree, it shows that the shortest path from unknown node *p* to direction anchor node *k* passes through target anchor node *i*. 

For the Equation (16), $Pc_{p,i}^{k}$ can be treated as the exponential function about the absolute cosine value of *angle*. The exponent $h_{p,k}/\left(h_{p,i}+h_{p,k}\right)$ shows the ratio of the hop number *h_p,k_* to the sum of hop numbers *h_p,i_* and *h_p,k_*. The smaller this ratio is, the closer the direction anchor node *k* is to the unknown node *p*, and then the calculated value of projection parameter is greater according to the property of exponential function. 

### 4.2. Weighted Processing for Correction Coefficients of All Direction Anchor Nodes

The correction coefficients for direction anchor nodes reflect their influence on the distance difference from unknown node to target anchor node. However, the correction coefficient only from a single direction anchor node cannot be sufficient to reflect the actual properties of the whole network. In addition, the influence of other direction anchor nodes are not fully utilized, which cause the phenomenon that the actual localization accuracy is not very high. 

The correction coefficients for all direction anchor nodes are considered in this paper, and the average weighted processing is employed to assign different weights to every correction coefficient respectively, and the final correction coefficient of the unknown node should be consisted of all the correction coefficients of direction anchor nodes, and it will be closer to the actual correction value in the network. The detailed equations for the average weighted processing are expressed as follows:

(18) $$w_{p,i}^{k}=\frac{h_{p,i}}{h_{p,k}}$$

(19) $${\bar{\omega }}_{p,i}^{k}=\frac{w_{p,i}^{k}}{\sum_{k}w_{p,i}^{k}}$$

(20) $$C_{p,i}=\sum_{k}{\bar{\omega }}_{p,i}^{k}\cdot C_{p,i}^{k}$$

where $w_{p,i}^{k}$, the ratio of *h_p,i_* and *h_p,k_*, represents the weight parameters for direction anchor node *k*, reflecting the distance relationship. If the ratio is relatively larger, it shows that the direction anchor node *k* is closer to the unknown node *p*, and then its weight parameter is greater. ${\bar{\omega }}_{p,i}^{k}$ is the normalized weight parameter for direction anchor node *k*, and the sum of all the normalized weight parameters for direction anchor nodes is ensured to be 1 through normalization. At last, *C_p_*_,*i*_ represents the final correction coefficient from the unknown node *p* to the target anchor node *i* after weight processing. 

### 4.3. Distance Correction

After the final correction coefficient from unknown node *p* to the target anchor node *i* is obtained, it can be used to correct the corresponding accumulated hop distance, so as to make the corrected distance closer to the actual Euclidean distance, and then the localization accuracy can be further improved. *Cdist_p_*_,*i*_ which represents the corrected distance from the unknown node *p* to the target anchor node *i* can be computed as follows:

(21) $$Cdist_{p,i}=\frac{d_{p,i}}{1+C_{p,i}\cdot h_{p,i}}$$

where *d_p,i_* and *h_p,i_* are respectively the accumulated hop distance and the hop number from unknown node *p* to target anchor node *i*. 

So far, after the unknown node gets correction distances from at least three anchor nodes, the position of unknown node can be computed using three-edge or multilateral distance method. 

## 5. Simulations and Performance Comparison

In order to verify the performance of the improved localization algorithm proposed in this paper, we use the simulation software MATLAB R2013a and compare the proposed algorithm with reference [21,22,23,24,25,26]. The reference [21] is the basic DV_Distance algorithm. References [22,26] respectively refer to the Dai’s method and the Shi’s method, and both of them use the ratio of actual Euclidean distance to accumulated hop distance to design correction coefficients, and references [23,24,25] employ the difference of actual Euclidean distance and accumulated hop distance. The Perkins’s method is in reference [23]; references [24,25] respectively describe the Liu’s method and the Wang’s method. 

**Figure 4:**
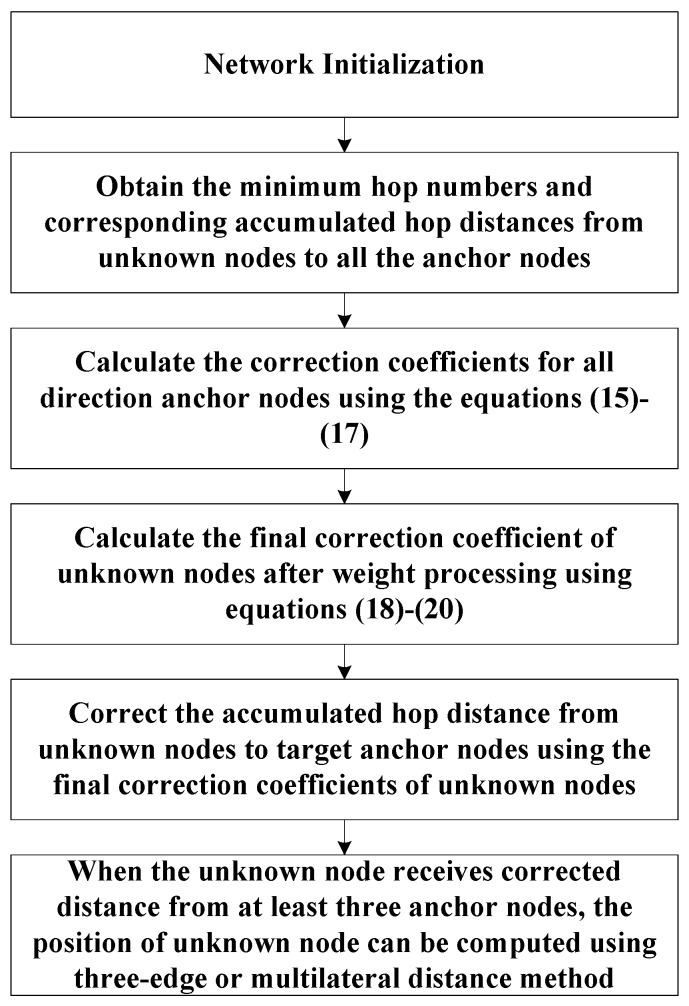
Simulation flow chart of the proposed algorithm.

Figure 4 shows the simulation flow chart of the proposed algorithm. 

### 5.1. Simulation Environment Requirement and Parameter Settings

#### 5.1.1. Simulation Environment Requirement

In order to objectively evaluate the performance of the improved algorithm and compare it with the existing algorithms, the simulation environment is set as follows:

(1) The network environment parameters are the same at each simulation running, such as the monitoring area of WSN, the total number of sensor nodes, the number of anchor nodes, and the communication radius of sensor nodes and so on;
(2) In the monitoring area of simulation, we randomly generate and deploy all the sensor nodes, and some sensor nodes randomly selected from sensor nodes are regarded as anchor nodes;
(3) The average error and the mean square deviation are respectively used to measure the accuracy and stability of localization, and the statistical result is based on independent simulation results of 100 times;

The equation for measuring localization accuracy is expressed as follows:

(22) $$\gamma =\frac{\sum_{i=1}^{Num}\sqrt{{\left(x_{i}^{true}-x_{i}^{est}\right)}^{2}+{\left(y_{i}^{true}-y_{i}^{est}\right)}^{2}}}{Num\cdot R}$$

where $\left(x_{i}^{true},y_{i}^{true}\right)$ is the actual position of unknown node *i*, and $\left(x_{i}^{est},y_{i}^{est}\right)$ represents the estimated coordinate of unknown node *i* calculated by localization algorithms. *Num* is the total number of unknown nodes, and *R* is the maximum communication radius of sensor nodes. 

The distance of adjacent nodes is determined according to the power of the received signal, and the influence of environment interference factors will cause some ranging error in the actual environment. In order to simulate this error, we use the product of the actual communication distance and Gauss white noise which standard deviation is *σ*. 

#### 5.1.2. Parameter Settings for Main Influence Aspects

(a) Parameter Settings for Influence Analysis of Ranging Error

In this situation, the standard deviation σ of Gauss white noise about ranging error gradually increases from 0 to 0.25, and the parameters in Table 2 are fixed. 

(b) Parameter Settings for Influence Analysis of Anchor Node Density

Under this case, the total number of anchor nodes is respectively, 15, 20, 25, 30, 35, and 40. Accordingly, the total number of unknown nodes is respectively, 85, 80, 75, 70, 65, and 60. The parameters in Table 3 are still unchanged for each independent running. 

(c) Parameter Settings for Influence Analysis of Maximum Communication Radius

In this condition, parameters in Table 4 are kept constant, and the maximum communication radius of sensor node is respectively set as 25 m, 30 m, 35 m, 40 m, 45 m, and 50 m. 

### 5.2. Results and Analysis

#### 5.2.1. The Influence Analysis of Ranging Error

**Figure 5:**
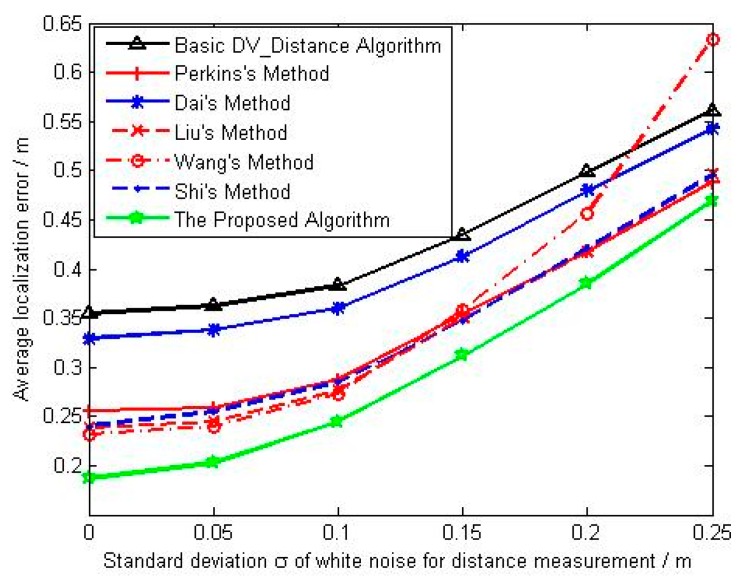
The change curve of average localization error with noise intensity.

**Figure 6:**
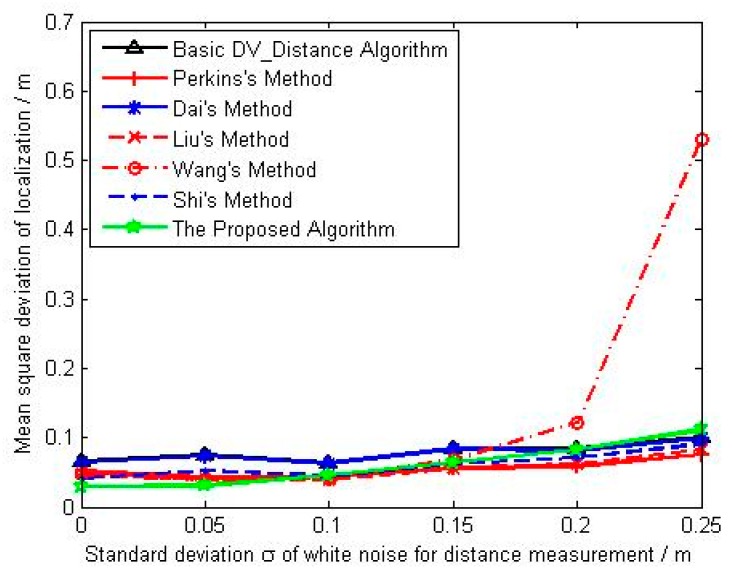
The change curve of mean square deviation with noise intensity.

It can be seen from Figure 5, the average localization error for each algorithm increases with the increase of noise intensity. The performance of basic DV_Distance algorithm is worst, and the main reason is that there is no correction on the accumulated hop distance. The ratio of the actual Euclidean distance to the accumulated hop distance is used by Dai, and we can see from the result that its average localization error is slightly better than that of basic DV_Distance algorithm. However, Perkins, Liu and Wang use the difference of the actual Euclidean distance and the accumulated hop distance, their localization error are obviously better than Dai’s method and basic DV_Distance algorithm. Liu makes the distance difference evenly distributed to each hop, and the average distance error per hop under unit distance is adopted by Wang. Under the low noise intensity, the average localization error of Wang’s method is slightly better than that of Liu’s method, and the average localization error of Liu’s method is also slightly better than that of Perkins’s method. With the increase of noise intensity, the average localization errors of Perkins’ method and Liu’ method tend to be the same, but the average localization accuracy of Wang’ method is significantly worse due to the great influence from noise. Similar to Dai’s method, the ratio of the actual Euclidean distance to the accumulated hop distance is used by Shi, but it uses the correction model based on unknown nodes, so its average localization error is better than basic DV_Distance algorithm and Dai’s method, but equivalent with the improved algorithms in references [23,24,25], which all use the distance difference to correct. The proposed algorithm combines the advantages of correction using distance difference and the correction model based on unknown nodes to calculate the correction coefficient of unknown nodes. From the results, we can see that the localization accuracy of the proposed algorithm is the best, and its performance is significantly better than the other algorithms. Figure 6 shows the change curve of mean square deviation with noise intensity. At the situation of low noise intensity, the stability of the proposed algorithm is better than that of other algorithms. With the increase of noise intensity, the localization stability of the proposed algorithm is basically equivalent to that of basic DV_Distance algorithm and methods in [22,23,24,26], and the localization stability of Wang’s method is the worst under high noise intensity. 

#### 5.2.2. The Influence Analysis of Anchor Node Density

**Figure 7:**
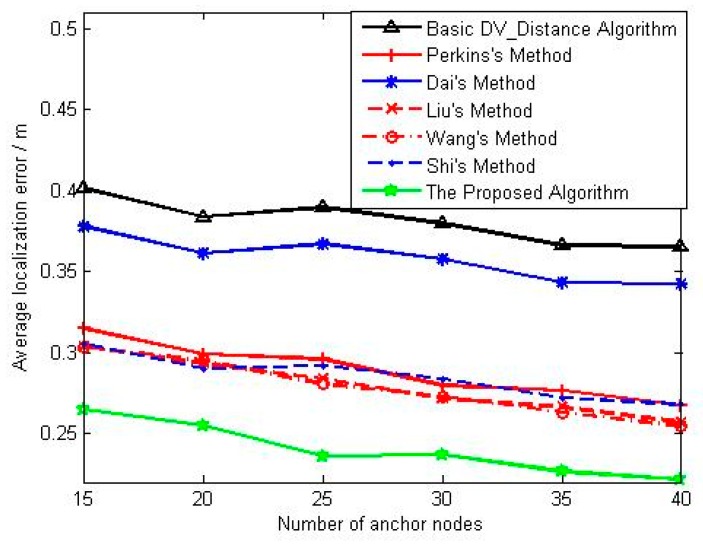
The change curve of average localization error with anchors density.

**Figure 8:**
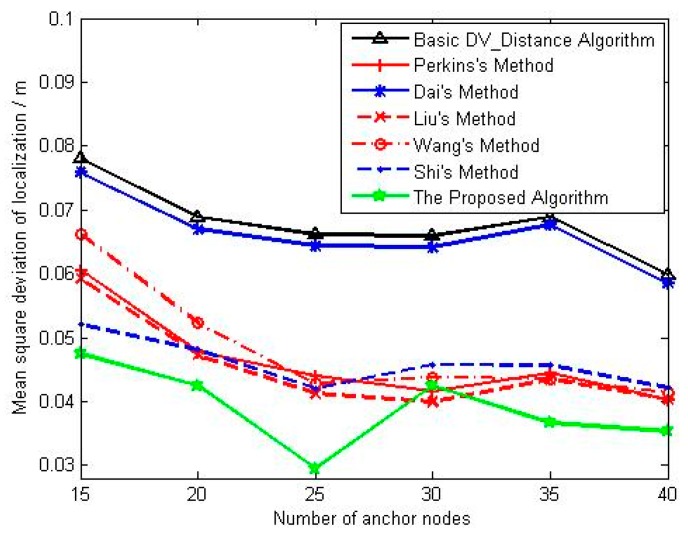
The change curve of mean square deviation with anchors density.

We can see that the average localization error and the corresponding mean square deviation decrease with the increase of anchors density. For the Figure 7, the localization accuracy of basic DV_Distance algorithm is still worst. Methods in references [23,24,25] are the improved localization algorithms using distance difference, and their localization accuracy is significantly better than that of Dai’s method. Besides that, the localization accuracy of Liu’s method and Wang’s method is also slightly better than that of Perkins’s method, and the main reason is that both Liu and Wang make the distance difference equally distributed to each hop. The localization accuracy of Shi’s method is similar to that of Liu’s method and Wang’s method in the situation of low anchors density, but is slightly worse than them with the increase of anchors density. The localization accuracy of the proposed algorithm is obviously better than that of other algorithms, that reason is mainly that the proposed algorithm not only uses the distance difference to correct, but also takes into account of the difference of correction coefficients from unknown nodes. For Figure 8, the localization stability of the proposed algorithm is better that of other algorithms, and the localization stability of methods in references [23,24,25,26] is better than that of basic DV_Distance algorithm and Dai’s method. Moreover, the localization stability of methods in references [23,24,25,26] will be consistent gradually with the increase of anchors density. 

#### 5.2.3. The Influence Analysis of Maximum Communication Radius

**Figure 9:**
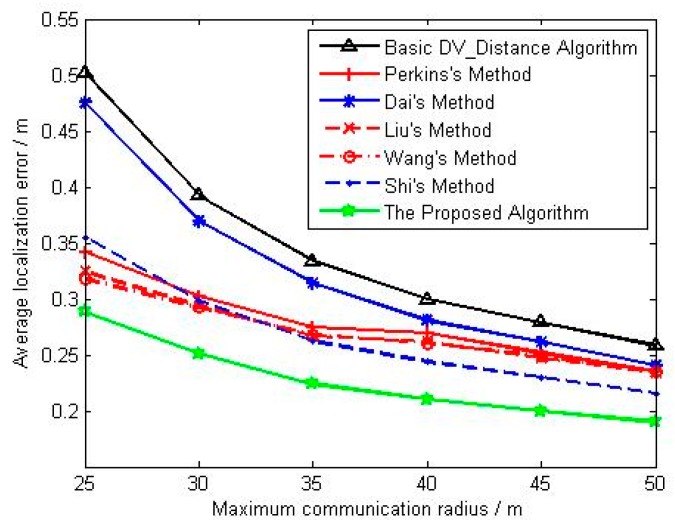
The change curve of average localization error with maximum communication radius.

**Figure 10:**
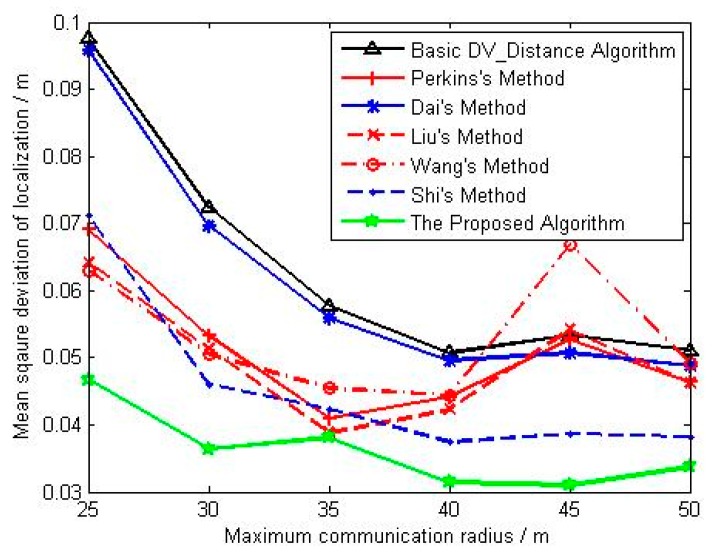
The change curve of mean square deviation with maximum communication radius.

Figure 9 shows the change curve of average localization error with maximum communication radius. The average localization error of all the algorithms decreases with the increase of the maximum communication radius, and the average localization accuracy of methods in references [23,24,25] is better than that of basic DV_Distance algorithm and Dai’s method, and their advantages are even more significant when the maximum communication radius is relative small. Although Shi uses the ratio of the actual Euclidean distance to the accumulated hop distance, the localization accuracy is slightly better than that of methods in references [23,24,25] with the increase of the maximum communication radius, and the main reason is that the correction model based on unknown nodes are made full use of in Shi’s method. The localization accuracy of the proposed algorithm is obviously better than that of other algorithms no matter what the maximum communication radius is. Figure 10 shows the change curve of mean square deviation with maximum communication radius. When the maximum communication radius is relatively small, the localization stability of methods in references [23,24,25] is superior to that of basic DV_Distance algorithm and Dai’s method. However, the localization stability of references [23,24,25] is gradually consistent with that of Dai’s method and basic DV_Distance algorithm with the maximum communication radius increasing. Besides that, when the maximum communication radius is relatively small, the localization stability of Shi’s method is the same as that of methods in references [23,24,25], but the localization stability of Shi’s method is better than them when the maximum communication radius is becoming large. Finally, the localization stability of the proposed algorithm is better than that of other algorithms no matter what the maximum communication radius is. 

To sum up, the average localization accuracy and stability of the proposed algorithm are better than those of other algorithms in each situation. 

## 6. Conclusions

In order to reduce the sensitivity of DV_Distance algorithms to ranging error and further improve the localization accuracy, a difference DV_Distance localization algorithm using correction coefficients of unknown nodes is proposed, and the proposed algorithm use the correction model based on unknown nodes, and the information from unknown nodes to anchor nodes is incomplete, so it cannot calculate the correction coefficient directly. The proposed algorithm adopts the difference of the accumulated hop distance and the actual Euclidean distance between anchor nodes to indirectly calculate the correction coefficients for different direction anchor nodes, and the final correction coefficients of unknown nodes can be obtained after weight processing according to their actual contribution, reflecting the difference of correction coefficients from different unknown nodes. The final simulation experiments demonstrate that the proposed algorithm has higher localization accuracy and the localization stability is also improved. 

## Figures and Tables

**Table 1:** Symbol description for some DV_Distance improvement algorithms.

Symbol	Description
i, j	Indexes for anchor nodes, *i* ≠ *j*;
k	Index for unknown node;
$h_{*,*}$	Hop number between sensor nodes;
$d_{*,*}$	Accumulated hop distance between sensor nodes;
$D_{i,j}$	Actual Euclidean distance from anchor *i* to anchor *j*;
$cv_{i,j}$	Correction coefficient from anchor *i* to anchor *j*;
$d_{k,j}^{est}$	Corrected distance from the unknown node *k* to anchor *j*;
$cv_{k,i,j}$	Correction coefficient from unknown node *k* to anchor *i* according to anchor *j*, *i* ≠ *j*;
$\omega_{k,i,j}$	Weighting factor for $cv_{k,i,j}$;
*N*	Total number of anchor nodes;

**Table 2:** Parameter table for influence analysis of ranging error.

Name	Value
Monitoring area	[0,100 m] × [0,100 m]
Total number of sensor nodes	100
Total number of anchor nodes	20
Total number of unknown nodes	80
Maximum communication radius	30 m

**Table 3:** Parameter table for influence analysis of anchor node density.

Name	Value
Monitoring area	[0,100 m] × [0,100 m]
Total number of sensor nodes	100
Maximum communication radius	30 m
Standard deviation *σ* of Gauss white noise	0.1

**Table 4:** Parameter table for influence analysis of maximum communication radius.

Name	Value
Monitoring area	[0,100 m] × [0,100 m]
Total number of sensor nodes	100
Total number of anchor nodes	20
Total number of unknown nodes	80
Standard deviation *σ* of Gauss white noise	0.1
